# Editorial: Reviews in neuro-otology: highlighting recent advances in neuro-otology: new possibilities for future inquiries

**DOI:** 10.3389/fneur.2025.1595204

**Published:** 2025-06-06

**Authors:** Philippe Perrin, Yoav Gimmon

**Affiliations:** ^1^Research Unit DevAH—Development, Adaptation and Handicap, Faculty of Medicine, University of Lorraine, Vandoeuvre-lès-Nancy, France; ^2^Laboratory for the Analysis of Posture, Equilibrium and Motor Function (LAPEM), and Department of Paediatric Oto-Rhino-Laryngology and Head and Neck Surgery, University Hospital of Nancy, Vandoeuvre-lès-Nancy, France; ^3^Department of Otolaryngology-Head and Neck Surgery, Sheba Medical Center, Tel Hashomer, Israel; ^4^Department of Physical Therapy, Faculty of Social Welfare and Health Sciences, University of Haifa, Haifa, Israel

**Keywords:** neuro-otology, cochlear implant, balance control, vestibular rehabilitation, vestibular function, vertigo, hearing loss

Neuro-Otology, a subspecialty of otolaryngology—head and neck surgery (also known as ear, nose, and throat/ENT medicine), is related to otology, clinical neurology and neurosurgery. Otology and neurotology are focused on the vestibular and cochlear systems and are sometimes considered together without a clear delineation between the two.

The vestibular system of the inner ear is responsible for the keeping balance and analyzing motion. This sensory organ enables us to find our bearings in space. It plays a key role in maintaining posture and stabilizing gaze.

Vertigo is the consequence of dysfunction of the afferents of the vestibular system (inner ear, vestibular nerve and central vestibular pathways) and sensory conflicts linked to the visuo-oculomotor and proprioceptive systems. Vertigo can force patients to modify their way of life. A diagnosis of vertigo allows for appropriate treatment to be proposed, which may involve medication, vestibular rehabilitation, surgery or psychotherapy. In particular, it is important to recognize that acute dysfunctions of the labyrinth can result in acute emergencies for the patient.

Vestibular rehabilitation, which concerns vertigo, dizziness and balance disorders, is based on the principle of using the plasticity of the central nervous system, i.e., its ability to develop new strategies to cope with damage to the peripheral vestibular system (1).

In this field, physiotherapists contribute to maintaining or restoring function, in particular through patient education and physical intervention. Vestibular rehabilitation involves a range of physical exercises, such as adaptation exercises to enhance vestibular function, substitution exercises to recruit an alternative strategy from additional systems (e.g., proprioception, vision), and habituation exercises for long-term reduction of response to noxious stimulus. In addition, instrumental maneuvers, using a rotating chair, optokinetic target generator, oculomotricity ramp, platforms (in particular, proprioception platforms), and virtual reality (which can be combined with posturography platforms), can also be used.

With regard to auditory function (the word audiology is from two roots: “Audio”, “to hear” and “logos,” “the study of”), hearing loss must be detected at birth, as delays in diagnosis can be detrimental to the child's development. During childhood, inflammatory rhinopharyngeal and otological disorders must be detected and managed by general practitioners, pediatricians, ENT specialists, school doctors and speech therapists. Hearing deficits related to acoustic trauma, tumor pathologies and syndromic disorders are managed by otologists. Excessive levels and duration of noise exposure (leisure activities, listening to music, workplace) can lead to inner ear hair cell damage. Presbycusis, which can be accompanied by tinnitus and hyperacusis, limits communication capabilities and can be a source of deterioration in quality of life, accompanied by neurocognitive disorders. This can result in social isolation and even depression. Technological advances have made hearing aids even more effective, requiring the expertise of an audiologist/hearing aid dispenser to adapt them to the individual's needs (2).

Of the eight articles accepted for this Research Topic, five concern hearing and three concern the vestibular system, reflecting the progress made in this field. Of the five hearing-related topics, three were on the topic of cochlear implants (CI), two discussed neuroinflammatory disorders and one reviewed health-related quality of life after otologic surgical treatment for chronic otitis media. In these review papers, the PRISMA statement was often adopted for the search strategy.

The systematic review of Schouwenaar et al. provides an overview of the evidence that otologic surgical treatment positively impacts Health-Related Quality of Life (HRQoL) among adult chronic otitis media patients with or without cholesteatoma, measured by various disease-specific HRQoL questionnaires. This evidence substantiates the importance of HRQoL assessment in clinical practice. However, the minimal clinically important differences between questionnaires have not been investigated yet, impeding drawing conclusions on clinical relevance. The systematic review included studies with various otologic surgical treatments and different HRQoL measurements. The review suggested that these diverse results could be generalized to adults suffering chronic otitis media with or without cholesteatoma, with regard to making decisions about surgical treatment. The authors point out that it is important to bear in mind that the fair study quality, intermediate risk of bias, and the high risk of selection bias indicate that actual outcome parameters might be less positive. Future research should also report primary preoperative symptoms and other indications for surgery to improve individual patient counseling (Schouwenaar et al.).

The results of the systematic review of Di Stadio et al. suggest that patients with migraine, multiple sclerosis, and Parkinson's disease have an increased risk of presenting with altered otoacoustic emissions compared to controls. From this perspective, and considering some shared neuro-inflammatory profiles, the authors speculate that pro-inflammatory cytokines could pass from the cerebrospinal fluid to the inner ear, with outer hair cell damage representing an early step in the sequential cascade of inner ear inflammatory disease. Transient-evoked otoacoustic emissions (detecting risk of hearing loss related to outer-hair-cell dysfunction) and perhaps even distortion product otoacoustic emissions responses (reflecting outer hair cell integrity and cochlear function) and their correlation with cerebrospinal fluid findings may help to elucidate active mechanisms of neuroinflammation during the subclinical phases of central nervous system neurodegenerative disorders. The authors hypothesize that the inner ear might be a novel anatomical entity from which the pathophysiology of brain diseases could be characterized more accurately, but it could also serve as a window of opportunity for early detection and treatment at a stage where the burden of such disorders is smaller and potentially reversible (Di Stadio et al.).

The optimal placement of a CI electrode inside the scala tympani compartment to create an effective electrode-neural interface is the basis of a successful CI treatment. The review carried out by Dhanasingh et al. sheds light on the requirements of an effective CI electrode array design which can correlate with overall variations of size, shape, and anatomy of the human inner ear, as well as minimizing intra-cochlear electrode insertion trauma. It is commonly agreed in the CI field that any degree of intra-cochlear trauma should be minimized during the electrode insertion process. Current scientific evidence indicates, at the time of writing of this article, that the straight lateral wall electrode outperforms the perimodiolar electrode type by preventing electrode tip fold-over and scalar deviation. Most of the literature comparing the hearing performance of perimodiolar and straight electrode types from the same CI manufacturer did not show the superiority of one electrode type over the other. However, facial nerve stimulation is reported to be minimized with perimodiolar electrodes as compared to the straight electrode type, a problem that is solvable by triphasic pulse stimulation or pseudo-monophasic stimulation with the latter. Almost three quarters of the scientific reports evaluating the effect of electrode angular insertion depth on hearing outcomes have confirmed increased hearing benefits associated with electrical stimulation covering both basal and middle turns of the cochlea. There are several explanations for this. Electrodes cover a broader frequency range and stimulate a larger number of neuronal cell bodies that are distributed up to 680° of angular insertion depth. In addition, there is a greater spatial separation between adjacent electrode contacts, and closer matching of electrodes to place-equivalent acoustic pitches. In summary, flexible and straight lateral wall electrodes are reported to be gentler to intra-cochlear structures and have the potential to electrically stimulate most of the neuronal elements. This all helps to maximize the benefits of the CI device to recipients (Dhanasingh et al.).

In the scoping review of CI users by Philips et al., it is highlighted that there is a need for guidelines on how listening effort, fatigue, and listening-related fatigue should be measured to allow for study results that are comparable and support optimal rehabilitation strategies. Research in these areas is in its early stages, while the number of CI users is growing. A standardized measurement method has not yet been developed for CI users or hearing-impaired groups (Philips et al.).

The study of Qin et al. aimed to assess the potential efficacy of cochlear implantation as a treatment for patients with Waardenburg syndrome (WS) and to guide clinical work by comparing the effect of auditory and speech recovery after cochlear implantation in patients with WS and non-WS syndrome. WS is an autosomal dominant genetic disorder primarily characterized by auditory pigmentary abnormalities. Its key manifestations include inner canthus heterotopia, iris heterochrony, white hair on the forehead, and hereditary sensorineural deafness (Qin et al.) (3).

Cochlear implantation demonstrates comparable auditory and speech recovery outcomes for WS patients and non-WS patients.

Not all dizziness presents as vertigo, suggesting other perceptual symptoms for individuals with vestibular disease. These non-specific perceptual complaints of dizziness have led to a recent resurgence in the literature examining vestibular perceptual testing, with the aim of enhancing clinical diagnostics and therapeutics. Recent evidence supports incorporating rehabilitation methods to retrain vestibular perception. The review of Grove et al. describes the current field of vestibular perceptual testing, from scientific laboratory techniques that may not be clinically friendly, to some low-tech options that may be more useful in the clinical setting. Limitations are highlighted and directions for future research are suggested (Grove et al.).

The work of Wolfovitz et al., encompassing the study of the vestibular system and associated disorders, shows the experienced notable growth and evolving trends over five decades. The changing landscape in vestibular science is outlined, focusing on epidemiology, peripheral pathologies, diagnosis methods, treatment, and technological advancements. The analysis underscored the dynamic nature of the field, highlighting shifts in focus and emerging publication trends in diagnosis and treatment over time. Etiologically, benign paroxysmal positional vertigo (BPPV) and Menière's disease were the most researched conditions, but the prevalence of studies on vestibular migraine showed a marked increase in recent years. Electronystagmography/Videonystagmography and Vestibular Evoked Myogenic Potentials were the most commonly discussed diagnostic tools, while physiotherapy stood out as the primary treatment modality. The analysis underscored the dynamic nature of the field, highlighting shifts in focus and emerging publication trends in diagnosis and treatment over time (Wolfovitz et al.).

The work of Cengiz et al. concerned the functional head impulse test, a non-invasive test that functionally evaluates the vestibulo-ocular reflex (VOR) of the six semicircular canals (4).

In clinical use, the determination of normative values at all accelerations (1,000–7,000 degrees/s^2^) constitutes an important data base for future studies to distinguish pathologic results. This study aiming to determine the normative values of the functional head impulse test in healthy young adults can be a guide for the vestibular rehabilitation process by evaluating different frequencies of the VOR arc in patients with balance problems.

By preparing exercise programs according to the affected VOR frequency, this will enable the targeted treatment process to begin. It can also be used for prognosis of disease and follow-up of the rehabilitation process (Cengiz et al.).

In particular, the authors of some of these *Reviews in neuro-otology* articles of this Research Topic have highlighted the need for additional research on these topics.

However, great strides have been made since it was thought that “Ear discharges were salutary for health, and that it is the bad humors that go out: they must not be dried up, because they would go elsewhere” and that “Perforated eardrums made you deaf” (5).
